# Low-Intensity Continuous Ultrasound vs. Traditional Ultrasound: A Systematic Review and Descriptive Analysis on Past and Future Gold Standards for Deep-Tissue Diathermy in Clinical Medicine

**DOI:** 10.3390/medsci14060631

**Published:** 2026-10-02

**Authors:** Samuel Wang, Aaditya Jain, Jemy M. Delikat, Jude Delikat, Samuel Phillips, Stephanie Petterson

**Affiliations:** 1 Clinical Research Department, Orthopaedic Foundation, Stamford, CT 06905, USA; samuelwangus@gmail.com (S.W.); aaditya.jain@uconn.edu (A.J.); 2 Research and Development Service, James A Haley Veterans Hospital, Tampa, FL 33612, USA; jemy.delikat@va.gov (J.M.D.); jude.delikat@hotmail.com (J.D.); samuel.phillips@va.gov (S.P.)

**Keywords:** therapeutic ultrasound, low intensity continuous ultrasound, sustained acoustic medicine, long duration ultrasound, diathermy, tissue temperature

## Abstract

Background/Objectives: The thermal effects of therapeutic ultrasound (US) have been utilized to promote musculoskeletal healing. However, the optimal combination of US parameters, while studied in the past, has yet to be updated with the advent of newer US modalities, including low-intensity continuous ultrasound (LICUS). The purpose of this study was to evaluate the effects of US frequency, intensity, and duration on changes in tissue temperature and to compare the thermal response between traditional US and LICUS. Methods: Following PRISMA guidelines, PubMed, Embase, and Web of Science were searched for in vivo human studies that reported temperature changes and used standard ultrasound parameters (1 or 3 MHz, 0.1–2.0 W/cm^2^, aqueous gel). US frequency, intensity, duration, effective radiating area (ERA), body region, and total energy delivered at three depths (1 cm, 3 cm, and 5 cm) were extracted. The outcome of interest was change in tissue temperature (i.e., ultrasonic diathermy). A mathematical model was used to interpolate missing data. Three-way ANOVA and t-tests were used to compare outcomes. Results: Nineteen studies (384 participants) were included. The weighted mean total energy delivered was 6698.5 ± 6767.4 J and was significantly correlated with tissue temperature change across all depths (r = 0.544, *p* < 0.001). At 1, 3, and 5 cm depths, significant interactions were observed between ultrasound intensity and frequency and between intensity and treatment duration (all *p* ≤ 0.05). Higher intensities generally produced greater temperature increases during shorter treatments (≤10 min), whereas longer treatment durations (≥60 min) at low intensity produced the greatest temperature increases overall. Frequency-dependent effects were also observed, with greater temperature increases at 3 MHz compared with 1 MHz at several intensity levels. LICUS delivered significantly more total energy than traditional 3 MHz US (13,854.4 ± 8129.6 vs. 3546.5 ± 2096.0 Joules, *p* < 0.001) and produced significantly greater tissue temperature increases at all depths (*p* < 0.001). Conclusions: Changes in tissue temperature are strongly correlated with the total US energy delivered. While frequency, intensity, and duration play a significant role in thermal energy, LICUS over sustained periods delivers significantly greater cumulative energy and, subsequently, greater tissue temperature change compared to traditional US.

## 1. Introduction

Therapeutic ultrasound (US) is a modality used to treat a wide variety of musculoskeletal conditions [1,2,3,4]. The thermal and mechanical effects of US create an environment that increases circulation, decreases inflammation, and promotes tissue repair [3,5,6,7]. Thermal therapeutic effects include pain reduction, accelerated metabolic rate, increased blood flow, increased extensibility of collagen fibers, and reduced viscosity of fluid elements in the tissue [1,2,3,4,5,7,8]. The mechanical effects of US include molecular vibration of tissue and the resultant friction, expansion and compression of tissue matrices, as well as acoustic streaming forces generated within tissues, leading to changes in cellular activity [9,10,11].

Three intrinsic parameters, frequency, intensity, and duration, can be manipulated to optimize therapeutic outcomes [12,13,14]. While studies have highlighted the influence of each parameter on depth of penetration and tissue-temperature increase [1,5,6,7,8,12,15,16,17], ambiguity remains regarding the optimal combination of these parameters, contributing to wide variability in clinical practice. Traditional, clinic-based, continuous US has inherent limitations, including short treatment duration, limited cumulative heating, and rapid post-treatment cooling [12,18,19,20].

Low-intensity continuous ultrasound (LICUS) has emerged as an alternative method of delivery designed to address these limitations, characterized by low intensity (0.1 W/cm^2^) and extended application durations ranging from 60 min up to 240 min [1,3,6,7]. In contrast to traditional US, which requires direct application by a healthcare provider in the clinical setting, LICUS is a prescription home-use, wearable treatment intended for prolonged use that provides uniform sustained deep-tissue heating over a large volume of tissue. LICUS devices have dual ultrasonic transducers with divergent 3 MHz continuous wave US emission to provide sustained acoustic medicine stimulation of tissue for 4 h without thermally overheating the targeted tissue.

While several healthcare policies and medical treatment guidelines exist for the use of traditional US and LICUS, no study has directly compared these two interventions in terms of tissue temperature increase. Therefore, the purpose of this study was to evaluate the thermal effects of US frequency, intensity, and duration on changes in tissue temperature and to compare thermal responses between traditional US and LICUS. The hypothesis was that LICUS would produce greater tissue temperature increases than traditional US.

## 2. Methods

### 2.1. Information Sources and Search Strategy

This systematic review and descriptive analysis was conducted in accordance with the Preferred Reporting Items for Systematic Reviews and Meta-Analyses (PRISMA) reporting guidelines and was registered with the Open Science Framework (OSF) Registry (https://osf.io/xzkp8, registered on 26 May 2026). A literature search was performed in PubMed, Embase, and Web of Science in October 2025. Search terms included combinations of ‘low-intensity continuous ultrasound’, ‘therapeutic ultrasound,’ ‘diathermic effects,’ ‘temperature change,’ ‘soft tissue,’ and related synonyms and MeSH terms. The search strings used for each database are included in Supplementary Table S1. All records were initially screened by title and abstract, followed by full-text review to determine eligibility by two independent reviewers (S.W. and A.J.). The remaining 19 studies were independently evaluated by both reviewers with a Cohen’s κ of 1, indicating complete agreement on their eligibility. Reference lists of included studies and relevant review articles were screened for additional studies. No restrictions were placed on the publication date.

### 2.2. Eligibility Criteria

Inclusion criteria were in vivo human studies published in English that reported temperature changes and US parameters. Data were included only from participants who received therapeutic US treatment with clearly defined and reported US parameters. Protocols encompassed traditional clinical US applications (i.e., aqueous coupling gel, conventional frequencies of 1 MHz or 3 MHz, and intensities ranging from 0.1 to 2.0 W/cm^2^), as well as LICUS delivery parameters (aqueous coupling gel, 3 MHz frequency, 0.132 W/cm^2^ intensity, treatment duration exceeding 60 min). Exclusion criteria were animal or in vitro studies, inability to obtain the data from the full-text article, studies examining cancer or other non-musculoskeletal conditions, studies using focused or pulsed ultrasound, and studies that did not report temperature changes or specific US parameters.

### 2.3. Data Extraction

Data from all included studies were extracted by two independent reviewers (S.W. and A.J.). No instances of duplicate or overlapping participant populations were identified. For each unique intervention arm or comparison, the following study characteristics were extracted: author(s), publication year, sample size, age, gender, heated body part, US frequency (MHz), US intensity (W/cm^2^), treatment duration (minutes/hours), and the effective radiating area (ERA). If the ERA was not reported, values were obtained from other studies utilizing the same US device or from the manufacturer’s manual. The primary outcome measure was the mean change in temperature at three depths: superficial (skin surface: 1 cm), intermediate (3 cm), and deep (5 cm).

A mathematical stepwise regression model was developed by the Department of Mathematics at Central Washington University to interpolate and fill in missing data points for comprehensive analysis. The model was built iteratively, adding variables (e.g., frequency, intensity, time and depth) that significantly improved the prediction of empirical temperature measurements. This initial model primarily used intensity as a predictor variable with a good fit to the extracted data at 1 cm (*p* = 0.031), 3 cm, (*p* = 0.025), and 5 cm (*p* = 0.019). Once the optimal set of variables was selected for correlation to empirical data, the resulting model was implemented into a Python script and used to estimate temperatures at depths where direct measurements were not available. These estimations were based on a thermodynamic exponential decay model and fit the expected exponential decay in ultrasonic absorption and heat transfer. This thermodynamic model was based on the equation:

(1) $$T\left(d\right)=Ae^{-rd}$$

where *T* is the temperature at a given depth *d*, A is the temperature at a depth of 0, and *r* is a coefficient that represents the decay rate of temperature as depth increases, which, in this model, was approximately 0.19. The total acoustic energy delivered was calculated using an established formula [21]:

(2) $$Total\;Energy\;\left(J\right)=Intensity\;\left(\frac{W}{cm^{2}}\right)\times ERA\;\left(cm^{2}\right)\times Duration\;\left(s\right)$$

### 2.4. Risk-of-Bias Assessment

The methodological quality and risk of bias for each study were assessed independently by two reviewers (S.W. and A.J.). The Cochrane Risk of Bias tool (RoB 2.0) was used to evaluate the risk of bias for randomized controlled trials (RCTs) [22]. Five domains were assessed: bias arising from the randomization process, bias due to deviations from intended interventions, bias due to missing outcome data, bias in the measurement of the outcome, and bias in the selection of the reported result. Risk of bias was categorized as no information, low, some concerns, or high risk.

The risk of bias for nonrandomized studies was evaluated using the Risk Of Bias In Nonrandomized Studies of Interventions (ROBINS-I) Version 2 tool [23]. ROBINS-I comprises seven domains and is scored similarly to the RoB 2 tool.

### 2.5. Statistical Analysis

Data analysis was performed using IBM SPSS Statistics (Version 30.0) or RStudio (Version 2026.05.0, Build 218). Descriptive statistics, including weighted means and standard deviations, were calculated for all continuous variables. Levene’s test was used to assess the homogeneity of variance (*p* > 0.05). A three-way univariate analysis of variance (ANOVA) was used to evaluate the effects of frequency (1 vs. 3 MHz), intensity (≤0.132 W/cm^2^, 0.5–1.0 W/cm^2^, and 1.5–2.0 W/cm^2^), and treatment duration (≤10 min, 20–60 min, and ≥60 min) on extracted and modeled tissue temperature changes. A separate analysis was conducted at each tissue depth to avoid pseudoreplication and to ensure that repeated modeled values from the same study would not be analyzed together. Tukey’s Honestly Significant Difference (HSD) tests were used for post hoc comparisons to minimize the risks of Type I error. Independent-samples t-tests were utilized to compare differences between LICUS and traditional US. A Pearson correlation coefficient was used to assess the relationship between total energy and tissue temperature changes.

To account for study-level clustering, a participant-weighted linear mixed-effects model was used in a sensitivity analysis. At each tissue depth, frequency, duration, and intensity were added as fixed effects, and the originating study was included to account for multiple observations originating from the same publication. Fixed effects were evaluated using Type III tests with a Satterthwaite approximation for denominator degrees of freedom. As an additional sensitivity analysis, data extracted at 1 cm were analyzed separately to assess whether the conclusions differed from those of the overall dataset. The 1 cm depth was selected because it had the largest number of data points. Similar to the original analysis, a Pearson correlation coefficient was calculated to assess the association between total energy and tissue temperature change. A three-way univariate ANOVA was used to assess the effects of frequency, intensity, and treatment duration on tissue temperature change. Due to the smaller dataset, intensity and duration were analyzed as continuous variables. Significance was defined as *p* < 0.05.

## 3. Results

The initial search identified 449 articles. Ultimately, 19 articles (*n* = 384 participants) met the inclusion and exclusion criteria and were included in the analysis. (Figure 1) Fifteen of the studies investigated traditional US [2,3,5,8,12,13,14,16,17,18,19,24,25,26] and five investigated LICUS [1,3,6,7,27], including one study that investigated both traditional US and LICUS [3]. There were 15 studies examining the calf [1,5,6,7,8,12,13,14,16,17,18,19,25,26,28], four in the forearm [1,3,6,27], one in the trapezius [2], and one in the Achilles tendon [24] (Table 1).

### 3.1. Risk of Bias Assessment

This review included one randomized placebo-controlled crossover study [27], one unblinded non-randomized comparative study [26], four randomized comparative or crossover studies [13,14,18,25], and 13 prospective comparative or repeated-measures studies [1,2,3,5,6,7,8,12,15,16,17,19,28]. The randomized controlled trial was classified as level I with a ROB-2 score of “no concerns” (Figure 2), whereas the remaining studies had a ROBINS-I of “low risk of bias” to “some concerns” (Figure 3). The overall risk of bias for nonrandomized studies was low to moderate (Figure 4).

### 3.2. Relationship Between Energy Input and Temperature Change

The ultrasound parameters and temperature increases from each study, as well as the modeled temperature changes, are summarized in Table 2. The weighted mean average of total energy delivered was 6698.5 ± 6767.4 J. There was a statistically significant correlation between the total energy delivered and tissue temperature change (r = 0.544, *p* < 0.001) across all depths (Figure 5).

### 3.3. Tissue Temperature Change at 1 cm Depth

There was a significant interaction between ultrasound intensity (low, medium, high) and frequency (1 MHz, 3 MHz) on tissue temperature change (*p* = 0.015), indicating that the effect of intensity on temperature change varied as a function of frequency. Post hoc analyses demonstrated that at 3 MHz, tissue temperature increase was significantly greater at high intensity (*p* < 0.001) and medium intensity (*p* < 0.001) compared to low intensity. There were no significant tissue temperature changes between medium and high intensity. At 1 MHz, the tissue temperature increase at high intensity was significantly greater than at medium intensity (*p* < 0.001). Additionally, at both high and medium intensities, the tissue temperature increase was significantly greater at 3 MHz compared with 1 MHz (*p* < 0.001 and *p* = 0.021, respectively; Figure 6a).

There was a significant interaction between treatment duration (≤10 min, 20–60 min, ≥60 min) and ultrasound intensity on tissue temperature change (*p* < 0.001), indicating that the effect of intensity on temperature increase was dependent on treatment duration. Post hoc pairwise comparisons demonstrated that, at ≤10 min, tissue temperature increases differed significantly across all intensity levels, with significantly greater tissue temperature increases at high versus medium (*p* < 0.001), high versus low (*p* < 0.001), and medium versus low (*p* < 0.001) intensities. At 20 to 60 min, tissue temperature increases were significantly greater at medium intensity compared to low intensity (*p* < 0.001). The greatest tissue temperature increase was seen with LICUS at low intensity with a treatment duration of 60 min or longer (Figure 7a).

At medium intensity, there was no significant difference between treatment durations of ≤ 10 min and 20–60 min. At low intensity, tissue temperature increases were significantly greater at 20–60 min compared to ≤ 10 min (*p* < 0.001), ≥60 min compared to ≤ 10 min (*p* < 0.001), and ≥ 60 min compared to 20–60 min (*p* < 0.001, Figure 7a).

### 3.4. Tissue Temperature Change at 3 cm Depth

There was a significant interaction between ultrasound intensity and frequency on tissue temperature change (*p* < 0.001). Post hoc analyses demonstrated that, at 3 MHz, tissue temperature increase was significantly greater at high intensity compared to low intensity (*p* < 0.01). There were no significant differences between medium and low intensity and medium and high intensity. At 1 MHz, the tissue temperature increase at high intensity was significantly greater than at medium intensity (*p* < 0.001). Frequency-dependent effects were also observed at medium intensity, with tissue temperature increases being significantly greater at 3 MHz compared with 1 MHz (*p* < 0.001). No significant difference was observed between 1 MHz and 3 MHz at high intensity (Figure 6b).

There was a significant interaction between treatment duration and ultrasound intensity (*p* < 0.001). Post hoc pairwise comparisons demonstrated that, at ≤10 min, a significantly higher tissue temperature increase was seen at high versus medium (*p* < 0.001), high versus low (*p* < 0.001), and medium versus low (*p* < 0.001) intensities. At 20 to 60 min, tissue temperature increase was significantly greater at medium intensity compared to low intensity (*p* < 0.001). The greatest tissue temperature increases occurred with LICUS at low intensity with a treatment duration of 60 min or longer (Figure 7b).

At medium intensity, temperature increase was significantly greater at treatment durations of 20–60 min compared to ≤10 min (*p* < 0.001). At low intensity, tissue temperature increases were significantly greater at ≥60 min compared to 20–60 min (*p* < 0.001) and ≤10 min (*p* < 0.001), as well as 20–60 min compared to ≤10 min (*p* < 0.001, Figure 7b).

### 3.5. Tissue Temperature Change at 5 cm Depth

There was a significant interaction between ultrasound intensity and frequency on tissue temperature change at 5 cm depth (*p* < 0.001). Post hoc analyses demonstrated that at 3 MHz, the tissue temperature increase was significantly greater at high intensity compared to medium intensity (*p* < 0.001) and low intensity (*p* < 0.001). There was no significant difference between medium and low intensity at 3 MHz. At 1 MHz, tissue temperature increases at high intensity were significantly greater than those observed at medium intensity (*p* < 0.001). At medium intensity, tissue temperature increases were significantly greater at 3 MHz compared with 1 MHz (*p* < 0.001). At high intensity, there was no significant difference in temperature increase between 3 MHz and 1 MHz (Figure 6c).

There was a significant interaction between ultrasound intensity and treatment duration on tissue temperature change (*p* < 0.001). Post hoc pairwise comparisons demonstrated that, at 10 min or less, tissue temperature increases were significantly higher at high versus medium (*p* < 0.001), high versus low (*p* < 0.001), and medium versus low (*p* < 0.001) intensities. At 20 to 60 min, there was no significant difference between low and medium intensities (Figure 7c).

At medium intensity, there was no significant difference between treatment durations of ≤10 min and 20–60 min. At low intensity, the tissue temperature increase was significantly greater at 20–60 min compared to ≤10 min (*p* < 0.001), at ≥60 min compared to ≤10 min (*p* < 0.001), and at ≥60 min compared to 20–60 min (*p* < 0.001, Figure 7c).

### 3.6. Sensitivity Analyses

The participant-weighted linear mixed-effects model resulted in ICC values between 0.39 and 0.5 across all tissue depths, indicating that 39%–50% of residual variance is attributable to between-study differences. As such, the ANOVA was re-analyzed using the results from the mixed-effects model to see if the conclusions of the study would change. The mixed-effects model ANOVA generally matched the results of the original ANOVA; there was only one comparison (duration at 5 cm, F = 2.58) that was originally significant, which became nonsignificant. The remainder of the duration comparisons at 1 cm and 3 cm, as well as the comparisons for intensity and frequency, retained their original significant/nonsignificant status, though the individual *p* values did change.

The sensitivity analysis restricted to the 1 cm extracted data showed trends similar to those observed in the overall analysis. Total energy delivered was significantly correlated with tissue temperature change (r = 0.523, *p* < 0.05, Figure 8a). In the three-way ANOVA, frequency was not significantly associated with temperature change, although only two data points were available at 1 MHz. The impact of intensity on temperature change approached significance (*p* = 0.059), whereas duration had a significant effect on temperature change (*p* < 0.01). When stratified according to the groups used in the original analysis, there was no significant difference between medium intensity and high intensity at ≤10 min (Figure 8b). Additional between-group comparisons could not be performed due to insufficient data with the remaining subgroups. 

### 3.7. Thermal Effects of LICUS vs. Traditional US

LICUS delivered significantly higher energy than traditional US at 3 MHz frequency regardless of treatment intensity. LICUS delivered more energy than both high intensity (13,854.4 ± 8129.6 J vs. 3936.4 ± 94.4 J; *p* < 0.001) and medium intensity (13,854.4 ± 8129.6 J vs. 3514.1 ± 106.9 J; *p* < 0.001) traditional US. Tissue temperature increases were significantly greater with LICUS compared to traditional US at 3 MHz frequency at all measured depths (*p* < 0.001, Table 3).

## 4. Discussion

LICUS delivered greater total energy and elicited significantly greater increases in tissue temperature compared to traditional US. While frequency and intensity also played a role in elevating tissue temperatures, the thermal effects were strongly dependent on exposure time, especially at lower intensities. LICUS had, on average, a larger ERA than traditional ultrasound, as well as a significantly longer exposure time, resulting in a large difference in cumulative diathermy dose between the two treatments (Figure 9). On average, traditional US provided approximately 3500 J of therapy in 10 min, while LICUS provided approximately 18,700 J across a 4 h treatment period. While traditional ultrasound causes a rapid initial increase in temperature, the tissue quickly cools down after treatment has stopped, resulting in less overall treatment than the consistently maintained elevated temperatures of LICUS therapy. Four hours after the start of therapy, the extracted and modeled tissue temperatures from traditional ultrasound have completely returned to baseline, while LICUS-induced temperature increases have just started to cool. LICUS also showed higher tissue temperatures than traditional ultrasound at similar depths across the modeled and extracted data points, which could be due to the sustained acoustic heating of LICUS offsetting the ambient cooling and temperature diffusion from blood flow occurring during therapy.

In addition to application duration, the significant interaction between ultrasound intensity and frequency highlights the role of acoustic attenuation. At superficial and intermediate depths (1 cm and 3 cm), 3 MHz was associated with higher extracted and modeled temperatures than 1 MHz at medium and high intensities. This aligns with the biophysical principle that higher frequencies are absorbed more rapidly, converting sustained acoustic energy into heat more effectively in the target tissue [12,29,30]. While no frequency-dependent difference was observed at the 5 cm depth at high intensity, this may reflect the inherent limitations of acoustic penetration at higher frequencies. The significant attenuation of 3 MHz waves in superficial layers likely resulted in a reduced data pool at deeper tissues, potentially limiting the statistical power to detect divergence between frequencies at this depth [14,16].

The interaction between treatment duration and intensity can be potentially explained by Pennes’ bioheat equation, which describes the balance between external energy delivery and the body’s natural cooling mechanisms [31]. While high-intensity US protocols typically produce rapid tissue temperature increases by overwhelming local tissue capacitance [8,28], temperature increases are counteracted by the body’s vasodilatory response, leading to heat dissipation. This physiological reaction, known as the “heat sink effect,” occurs to facilitate rapid heat dissipation via increased blood perfusion. In contrast, LICUS utilizes a constant, lower rate of heating over an extended period of time to achieve a slower increase in temperature and higher overall temperature increases [3,6]. This allows energy to slowly accumulate in tissue without triggering a rapid cooling response by the circulatory system [32]. When low-intensity US is applied for durations greater than 60 min, cumulative energy accumulation eventually outpaces the thermal dissipation by local blood flow, leading to a slow, sustained increase in tissue temperature to maximize thermal and mechanical therapeutic effects [2,17].

Total energy dose is critical in maximizing the increase in tissue temperature. LICUS maintains energy delivery for prolonged periods, resulting in a four-fold higher total energy dose than traditional US [1]. This sustained delivery allows the energy to saturate the tissue, effectively overcoming the heat sink effect and providing more effective deep-tissue heating, even while operating at a much lower intensity [7]. LICUS also increases local blood circulation, promoting tissue oxygenation, metabolic activity, and cellular migration, all of which are essential for the modulation of inflammation and the repair of damaged muscle tissue [7,27].

Clinically, several recent studies and systematic reviews have been completed on LICUS and traditional ultrasound. Mohammed et al. found moderate to high methodological quality evidence that traditional US and exercise provided improvements in pain and function for patients with knee osteoarthritis [33]. Guan et al. concluded that the current clinical evidence for US therapy supports its effectiveness in reducing pain and aiding rehabilitation for knee conditions [34]. Uddin et al. reviewed the broad, evolving field of home-use LICUS-based treatments and its implications in both pre-clinical and clinical applications [35]. Furthermore, Winkler et al. specifically reviewed the clinical evidence on 4 h LICUS treatment of musculoskeletal conditions and determined that it provided vigorous diathermy of Δ4 °C to Δ12 °C, significantly reduced joint pain, and improved overall joint function [36]. Although LICUS and traditional US were effective as conservative and non-opioid treatment options for joint pain and knee osteoarthritis, US exposure parameters are critically important to generate deep heat at therapeutic levels for desired clinical outcomes.

The delivery of US at sub-therapeutic levels is not clinically helpful, as described by Alexander et al. [37]. The authors found that, across eight studies and *n* = 586 patients, US administered to deliver at least 4228 J per treatment provided significant benefit to patients with shoulder pathologies. However, US treatments with less than 2019 J showed only limited to no benefit. Delivery of 720 J per treatment or less showed no improvement in any study identified. Based on the work of Alexander et al., the total ultrasound energy delivered by LICUS or traditional ultrasound is an important indicator for both deep-tissue heating and clinical response to treatment.

The studies included in this analysis showed varying levels of ultrasound energy delivered per treatment, ranging from 147 J to 19,720 J, with a direct correlation to increased tissue temperature rise. Interestingly, only five of the twenty-three traditional US studies included (21.7%) met the Alexander et al. [37] therapeutic benefit threshold of greater than 4228 J delivered per treatment. For LICUS treatment, four of five studies (80%) met this therapeutic benefit threshold. For multi-hour sustained acoustic medicine, a subset of the LICUS group, 100% of studies exceeded the therapeutic threshold, delivering ≥ 4228 J per treatment. The current analysis shows that LICUS treatment delivers both greater total energy and tissue temperature increases than traditional US. For both clinicians and payer policy decision makers, LICUS-based treatment strategies should be considered and covered for patients where traditional US is indicated. Finally, the emergence of high-dosimetry LICUS home-use-based treatment options has opened a new treatment approach for pain management and rehabilitation medicine.

One important point of discussion is the safety of LICUS devices, especially given the large increase in delivered energy with longer treatment times. It is established that significantly elevated tissue temperatures, e.g., from focused ultrasound, can lead to cellular damage [38]. However, LICUS devices typically use divergent beam ultrasound, which disperses energy over a larger area, providing a lower magnitude of heating that does not reach a damaging threshold. This is also reflected in the published literature. Across numerous clinical trials that included a total of 468 participants, no adverse events related to ultrasound treatment were reported [27,39,40,41,42,43,44,45]. This includes studies where LICUS devices were given to patients for at-home use, indicating that these devices can be used by non-trained personnel outside of the clinic without causing any treatment-related adverse events.

Because this study used both extracted and modeled data, there is some degree of uncertainty inherent to the estimated temperature changes seen here, particularly when modeled data were used. However, several features of the included studies may mitigate potential variability in the extracted measurements. Most studies, including the non-randomized studies, used baseline temperatures from the same subject or temperatures from the opposing limb as a control, thereby reducing the confounding effects of between-subject variability. In addition to this, temperature was measured at consistently defined depths using electronic temperature sensors that were usually calibrated and/or verified during the study, reducing the likelihood of bias from the measurement methods. When the extracted data at 1 cm was analyzed alone, the findings were consistent with the primary analysis. Total energy delivered was consistently correlated with temperature change, and treatment duration played a role in the overall temperature change. Meanwhile, intensity demonstrated a weaker relationship. Because basic thermodynamic principles of ultrasound indicate an exponential dissipation of heat in tissue, it is anticipated that trends at 3 cm and 5 cm would be similar to those seen at 1 cm. Nevertheless, greater uncertainty remains regarding the magnitude of temperature change at 3 cm and 5 cm depths, and these estimates should therefore be interpreted with appropriate caution.

While both traditional US and LICUS use the same energy form—ultrasound—to deliver treatment, they differ in their treatment parameters and mode of administration. Traditional US is typically delivered in a clinical setting at higher intensities over a relatively short treatment duration [2,29,37], while LICUS uses a lower intensity over a substantially longer duration and is designed for self-administration and home use. The lower intensity of LICUS may suggest a reduced potential for tissue heating compared to clinically administered ultrasound. However, the results seen in the present study show that the longer duration of LICUS treatment offsets the lower intensity through cumulative energy delivery, resulting in meaningful increases in tissue temperature. These findings suggest that LICUS can achieve tissue heating despite its lower treatment intensity while providing the practical advantage of prolonged, home-based ultrasound delivery.

This analysis has several inherent limitations. First, the included studies utilized devices from different manufacturers and utilized varying coupling media, introducing variability that may impact energy transmissibility. Second, mathematical modeling was required for missing data points in some studies to allow for a standardized comparison across depths, which may introduce estimation error. Third, many of the included studies had limited sample sizes and were not published recently. More recent studies in the literature focus on clinical outcomes, such as pain and function changes, rather than ultrasonic dose and temperature change. However, it is still valuable to compare devices based on their ultrasound parameters to examine an objective outcome like temperature change regardless of the age of the devices, since the underlying technology for therapeutic ultrasound is well-established. The process used here could very easily be expanded to include newer studies that examine tissue diathermy, and a future analysis could consider combining ultrasonic dose, temperature change, and clinical outcomes to better understand the dose–response relationship.

An additional limitation of this analysis is the impact of the underlying tissue on the diathermic effect. Because various tissues can have different levels of subcutaneous fat and vascularization, and different tissue compositions can attenuate ultrasonic waves differently [46], a portion of the variation in temperature change seen here could be explained by variations in body composition rather than differences in total applied energy. There could also have been variations in body composition across all the participants in the included studies which could influence the comparisons discussed here. Because there were multiple regions of the body being compared, tissue compositional differences may have influenced the pooled comparisons and reduced the generalizability of these results. Future studies could directly examine therapeutic ultrasound and LICUS devices in the same cohort in the same body region to better elucidate the differences in efficacy between these two treatment methods. The studies selected for this analysis were also restricted to English-language studies, which could have removed data that could improve the model. Finally, while tissue temperature and total energy delivered are good measurements of physiological change and the purpose of diathermy, this analysis did not directly evaluate the relationship between temperature increase and clinical outcomes. Additional research combining the work of Alexader et al. [37] and this study could guide clinicians in standardizing therapeutic and sub-therapeutic ultrasound dosing for desired clinical outcomes.

## 5. Conclusions

Changes in tissue temperature are strongly correlated with the total US energy delivered. While frequency, intensity, and duration play a significant role in thermal energy, low-intensity continuous US delivers significantly greater cumulative energy over sustained periods and subsequently greater tissue diathermy and overall temperature change compared to traditional US. Multi-hour LICUS-based treatment options should be considered as a readily accessible home-based care option for patients when traditional US diathermy is indicated. Future work should compare pain and functional outcomes between both therapies to determine which option is more appropriate to heal the patient.

## Figures and Tables

**Figure 1:**
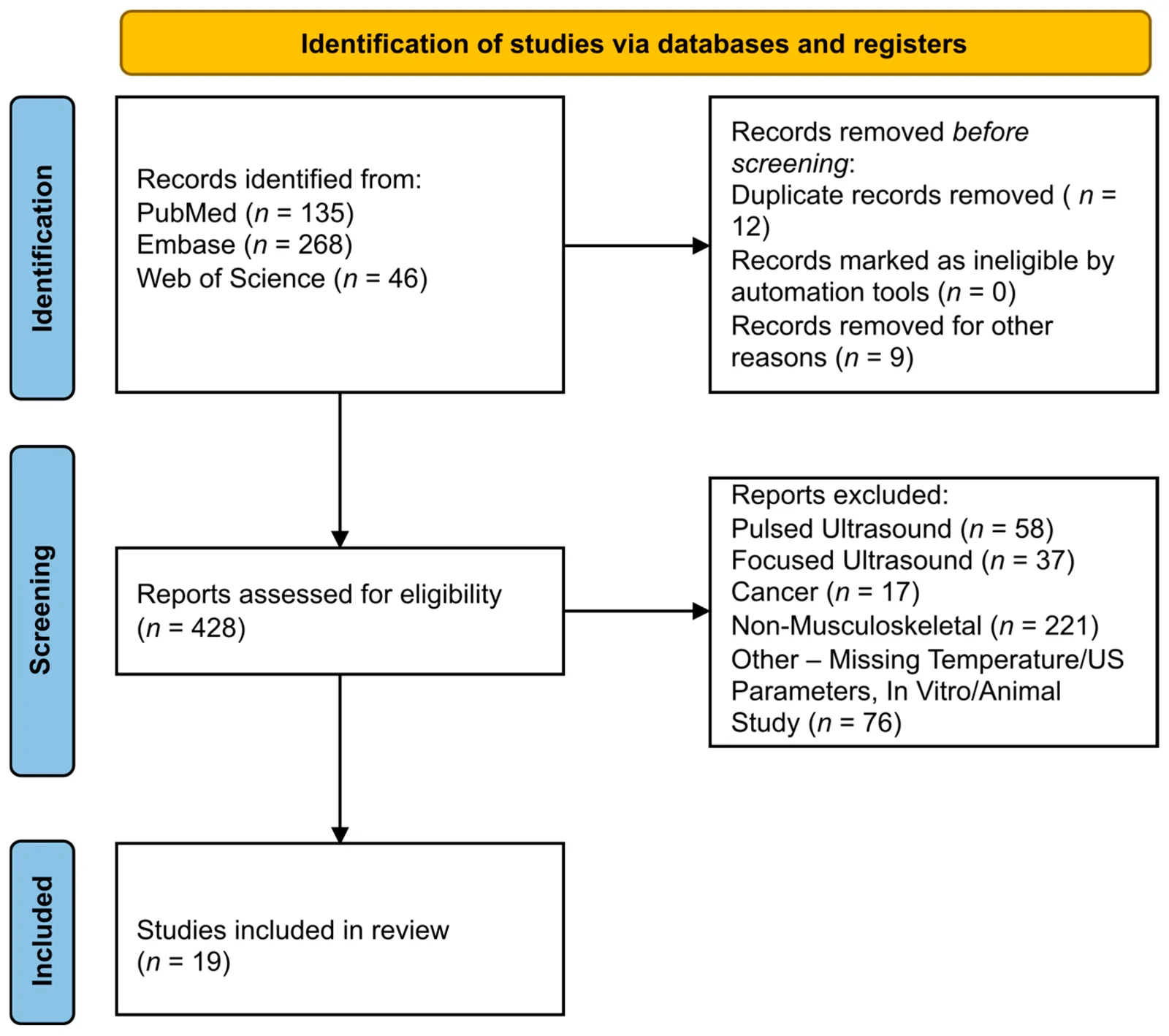
PRISMA flow diagram of included studies.

**Figure 2:**
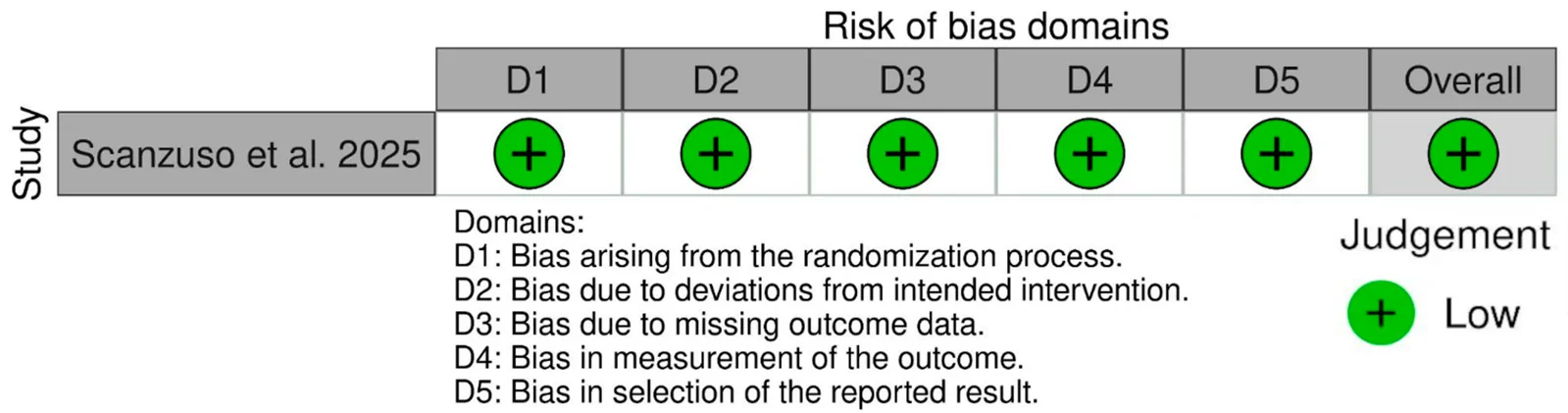
Risk of bias for randomized controlled trials using the Cochrane Risk-of-Bias 2 tool [22,27].

**Figure 3:**
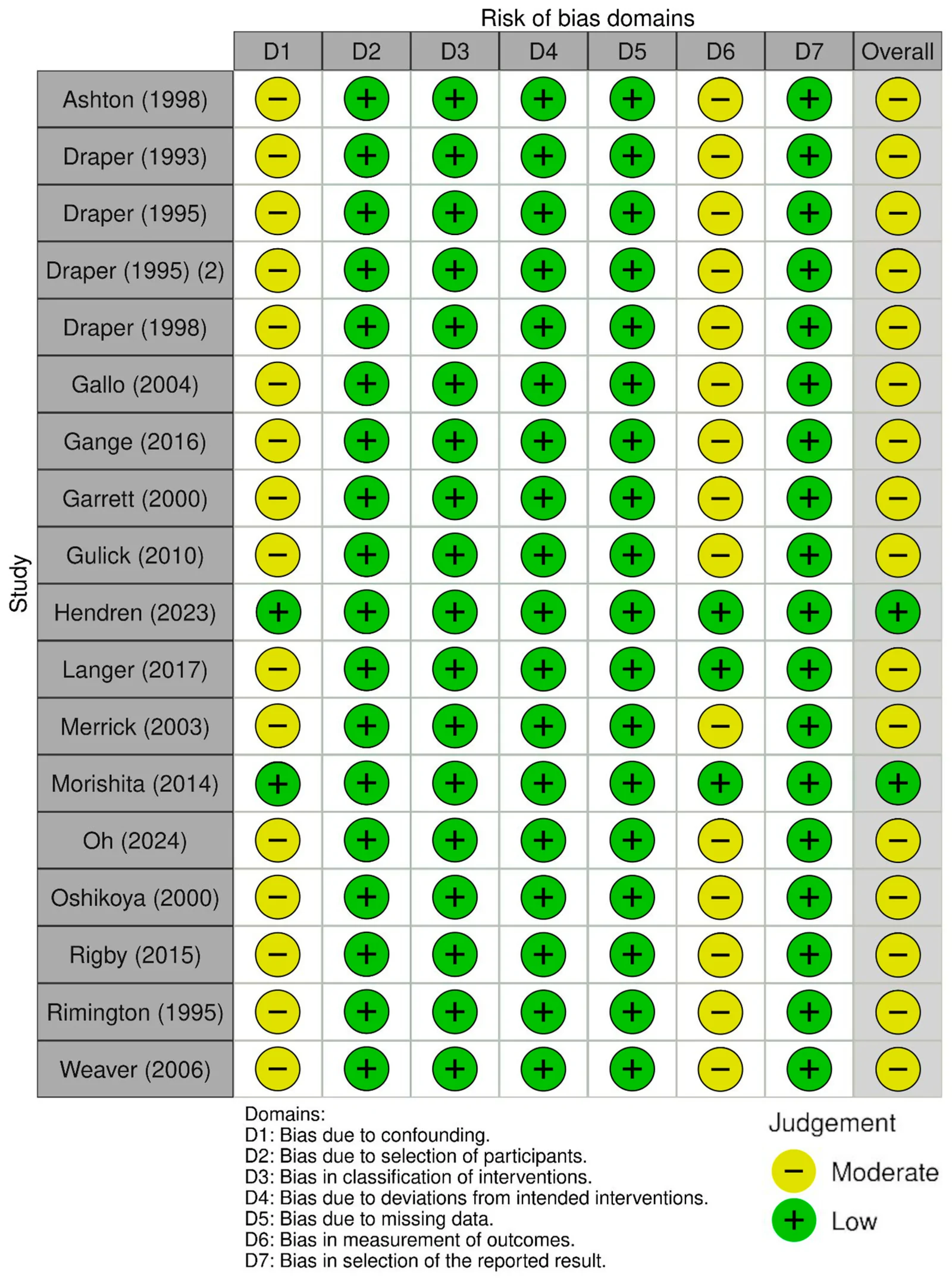
Risk of bias for nonrandomized controlled trials using the Cochrane Risk Of Bias In Non-randomized Studies of Interventions tool [1,2,3,5,6,7,8,12,13,14,15,16,17,18,19,23,25,26,28].

**Figure 4:**
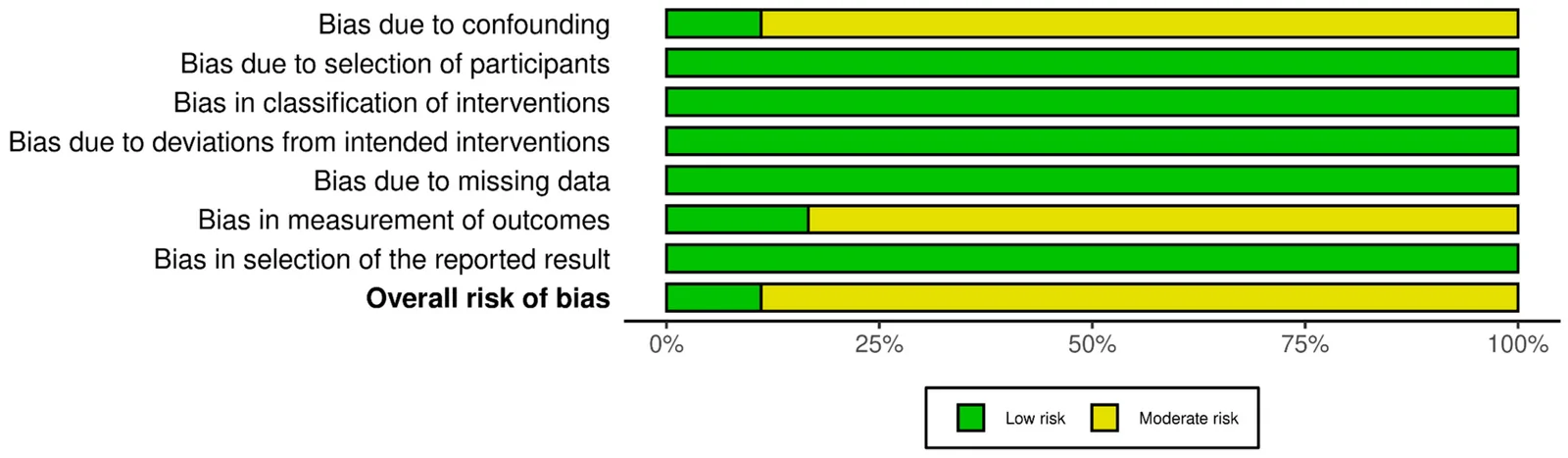
Risk-of-bias summary for all nonrandomized studies using the Cochrane Risk Of Bias In Non-randomized Studies of Interventions tool.

**Figure 5:**
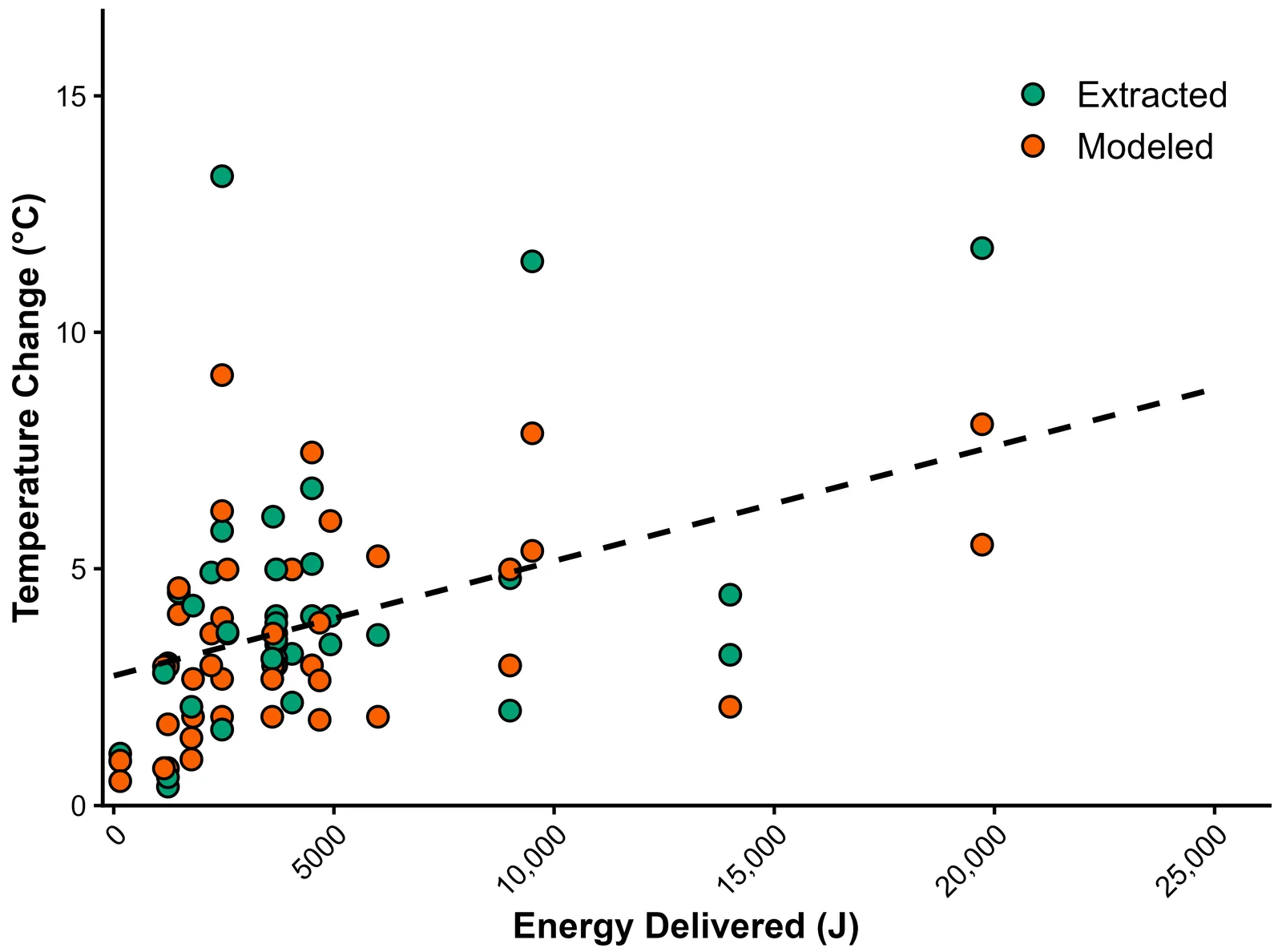
Correlation between energy delivered and temperature change for traditional ultrasound and low-intensity continuous ultrasound therapies. Dashed line represents line of best fit.

**Figure 6:**
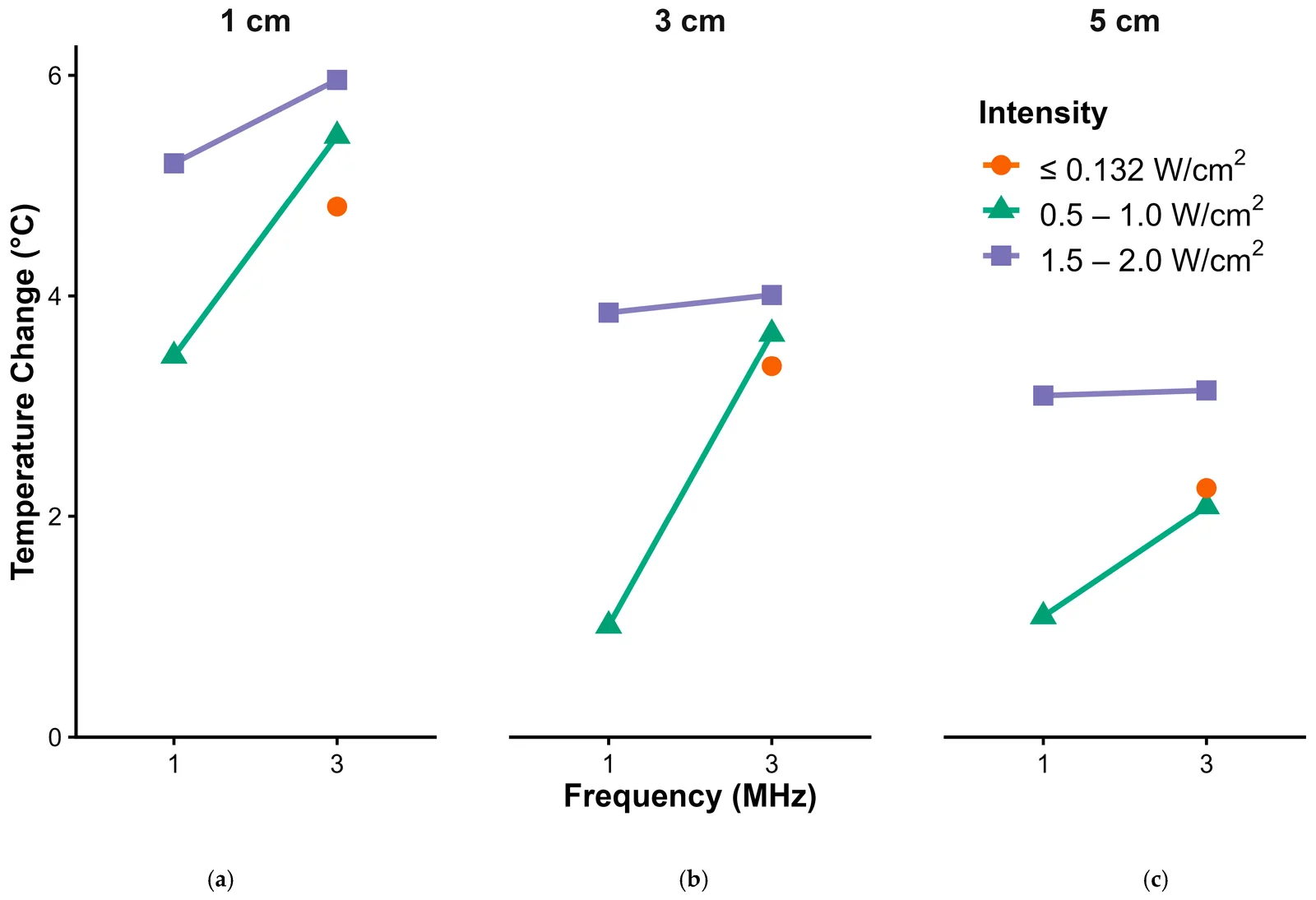
Mean temperature increase as a function of frequency at (**a**) 1 cm, (**b**) 3 cm, and (**c**) 5 cm tissue depths.

**Figure 7:**
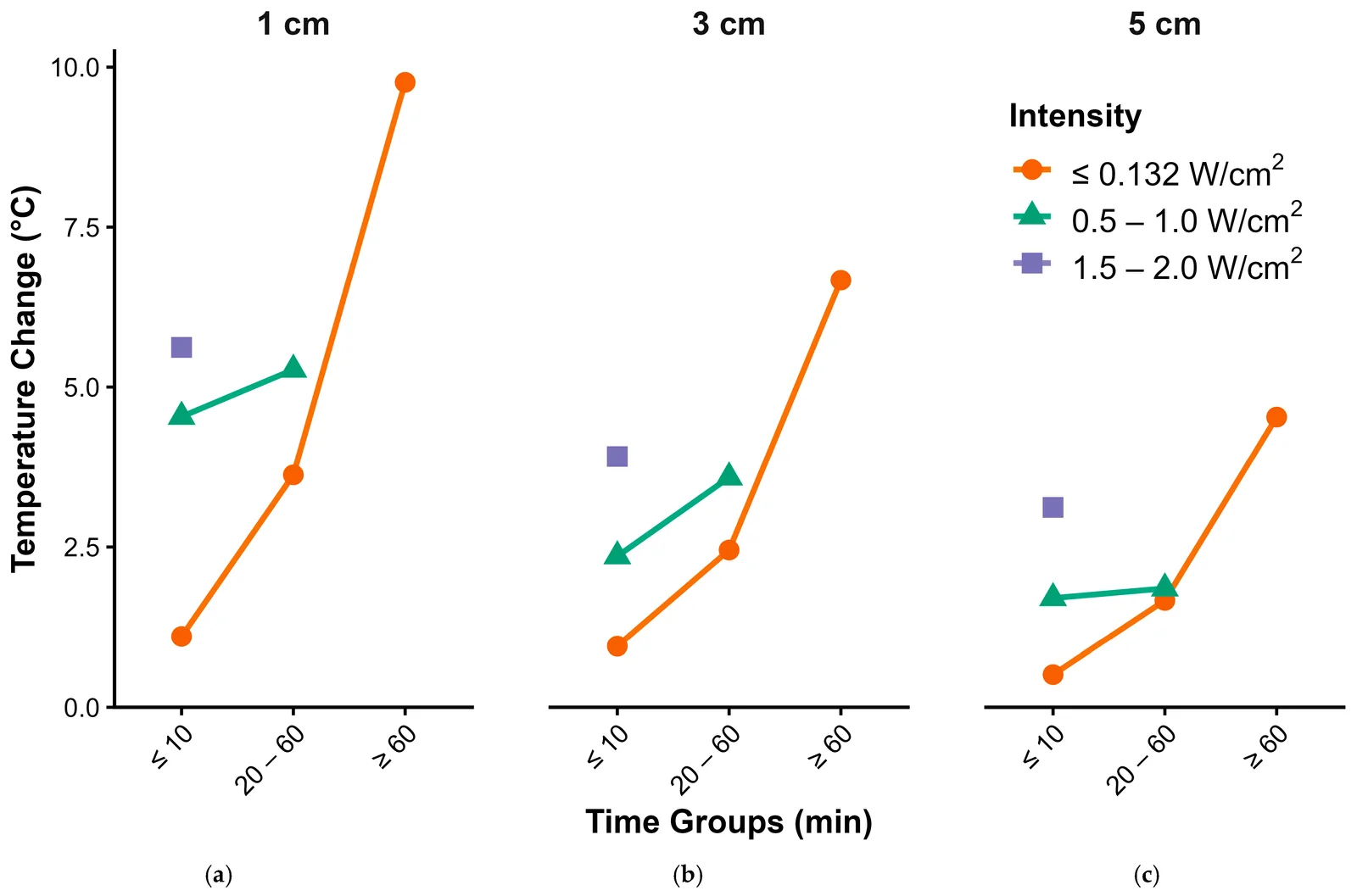
Mean temperature increase as a function of treatment duration at (**a**) 1 cm, (**b**) 3 cm, and (**c**) 5 cm tissue depths.

**Figure 8:**
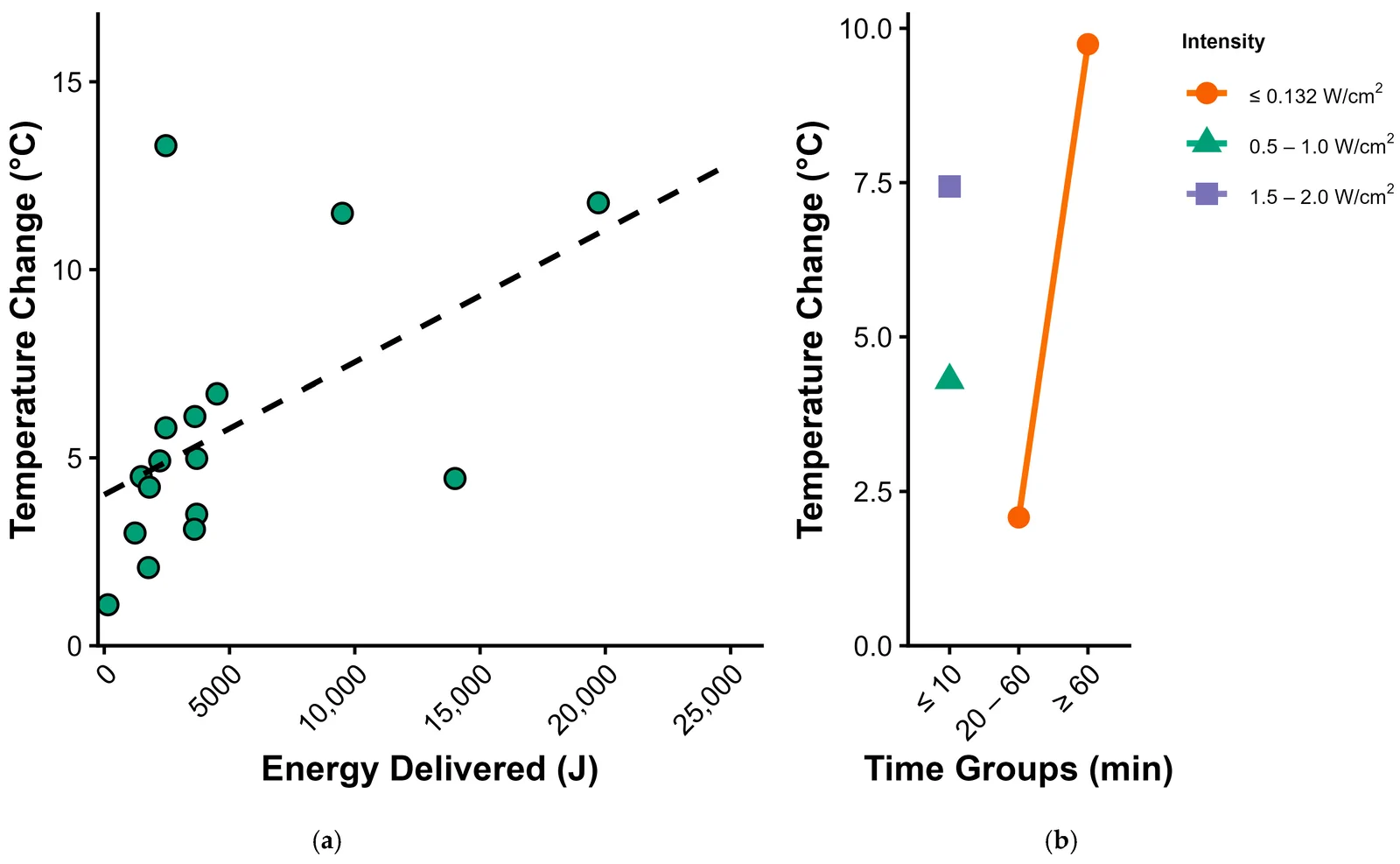
(**a**) Correlation between temperature change and energy delivered for extracted data at 1 cm. (**b**) Mean temperature increase as a function of treatment duration for extracted data at 1 cm.

**Figure 9:**
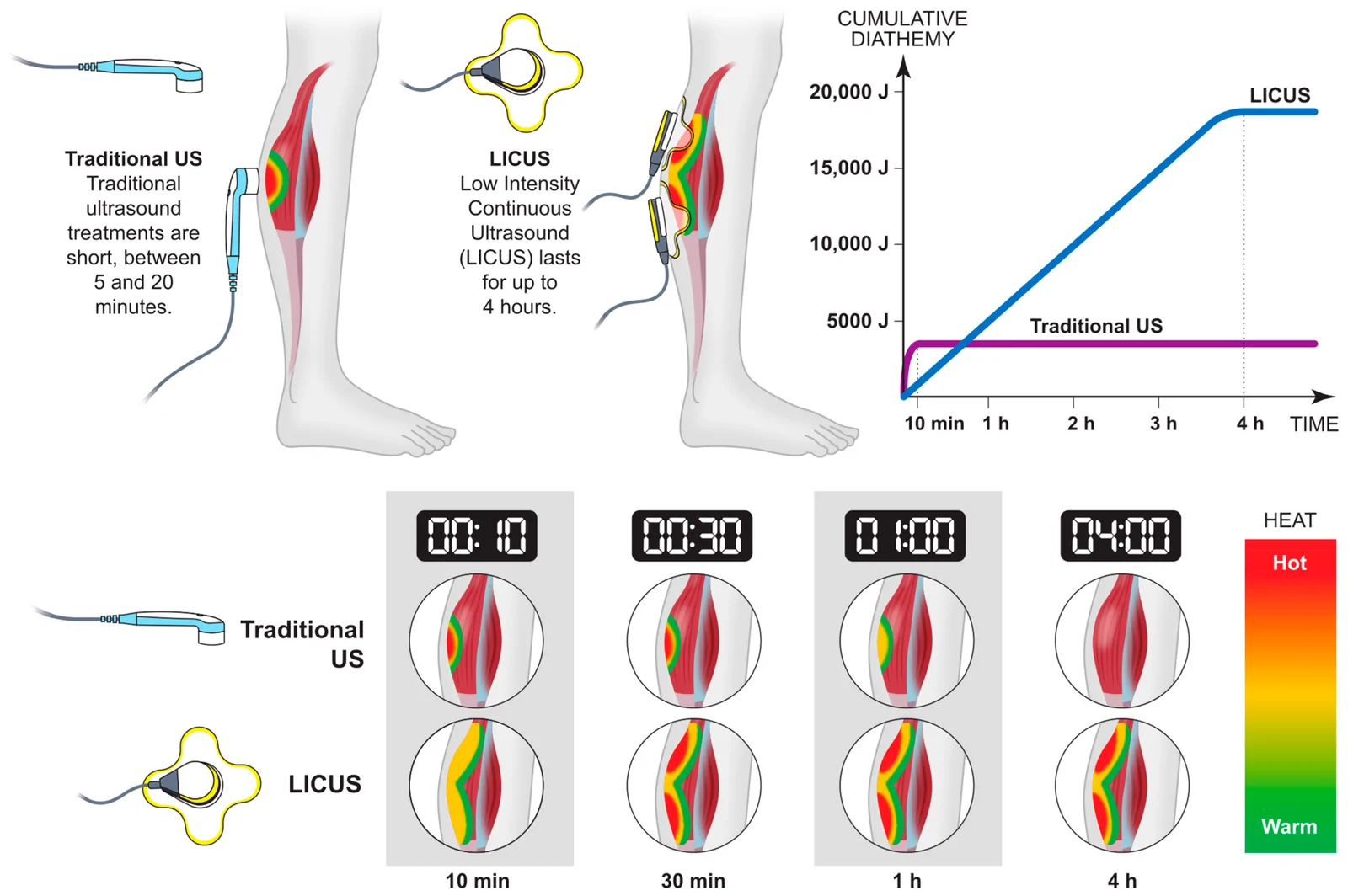
Comparison between traditional ultrasound (US) and low-intensity continuous ultrasound (LICUS) in cumulative diathermy and temperature increase over time.

**Table 1:** Participant demographics of included studies.

Article (Author, Year)	Number of Participants	Gender, N (Male/Female)	Age, Mean Years (SD or Range)	Body Part
Ashton, D.F., 1998 [16]	15	N/S	24.2 (3.7)	Calf
Draper, D.O., 1993 [14]	20	20 / 0	23.0 (19–31)	Calf
Draper, D.O., 1995a [12]	12 ^†^	N/S	22.0 (1.4)	Calf
Draper, D.O., 1995b [13]	8 ^†^	N/S	26.0 (21–37)	Calf
Draper, D.O., 1998 [18]	9 ^†^	N/S	23.7 (1.7)	Calf
Gallo, J.A., 2004 [25]	16	5/11	21.3 (2.5)	Calf
Gange, K.N., 2016 [5]	30	19/11	21.3 (2.0)	Calf
Gulick, D.T. 2010 [26]	40	14/26	23.8 (3.1)	Calf
Merrick, M.A., 2003 [8]	6	4/2	22.0 (3.4)	Calf
Morishita, K., 2014 [2]	3	3/0	25.0 (20–39)	Trapezius
Oh, S., 2024 [3]	10	N/S	23.15 (2.3)	Forearm
Oshikoya, C.A., 2000 [17]	18	18/0	23.6 (3.5)	Calf
Rimington, S.J., 1995 [19]	8 ^†^	8/0	24 (1.6)	Calf
Weaver, S.L., 2006 [28]	11	9/2	22.6 (1.7)	Calf
Draper, D.O., 2010 [24]	40 ^†^	20/20	21.5 (1.5)	Achilles Tendon
Hendren, T.F., 2023 [1]	14 ^†^	N/S	18.0 (18–65)	Calf and Forearm
Langer, M.D., 2017 [6]	44	N/S	N/S	Calf and Forearm
Rigby, J.H., 2015 [7]	20 ^†^	N/S	23.0 (2.1)	Calf
Scanzuso, A., 2025 [27]	60 ^†^	N/S	24.85 (9.1)	Forearm

^†^ = Some participants excluded based on exclusion criteria; N/S = not specified.

**Table 2:** Ultrasound parameters and changes in temperature for traditional and low-intensity continuous ultrasound.

Article (Author, Year)	Frequency (MHz)	Intensity (W/cm^2^)	Time (min)	Effective Radiating Area (cm^2^)	Total Energy (J)	Temp Rise at 1 cm	Temp Rise at 3 cm	Temp Rise at 5 cm
Traditional Ultrasound								
Ashton, D.F., 1998 [16]	1	1.5	10	4.5	4050	4.985 ^†^	3.200	2.170
Draper, D.O., 1993 [14]	1	1.5	10	10	9000	4.985 ^†^	4.800	2.955 ^†^
Draper, D.O., 1995a [12]	3	0.5	10	4.1	1230	3.000	1.710 ^†^	0.785 ^†^
Draper, D.O., 1995a [12]	3	1	10	4.1	2460	5.800	2.670 ^†^	1.870 ^†^
Draper, D.O., 1995a [12]	3	1.5	6	4.1	2214	4.920	3.630 ^†^	2.955 ^†^
Draper, D.O., 1995a [12]	3	2	3	4.1	1476	4.500	4.590 ^†^	4.040 ^†^
Draper, D.O., 1995a [12]	1	0.5	10	4.1	1230	2.935 ^†^	0.400	0.600
Draper, D.O., 1995a [12]	1	1	10	4.1	2460	3.960 ^†^	1.600	1.600
Draper, D.O., 1995a [12]	1	1.5	10	4.1	3690	4.985 ^†^	3.400	3.100
Draper, D.O., 1995a [12]	1	2	10	4.1	4920	6.010 ^†^	4.000	3.400
Draper, D.O., 1995b [13]	1	1.5	10	4.1	3690	4.985	3.630	4.000
Draper, D.O., 1998 [18]	1	1.5	10	4.1	3690	3.500	3.850	2.955 ^†^
Gallo, J.A., 2004 [25]	3	0.5	10	3.8	1140	2.935 ^†^	2.800	0.785 ^†^
Gange, K.N., 2016 [5]	3	1	6	5	1800	4.220	2.670 ^†^	1.870 ^†^
Gange, K.N., 2016 [5]	3	1	20	5	6000	5.264 ^†^	3.600	1.870 ^†^
Gulick, D.T., 2010 [26]	3	1.5	10	5	4500	6.700	4.000	2.955 ^†^
Merrick, M.A., 2003 [8]	3	1.5	6	6.7	3618	6.100	3.630 ^†^	2.955 ^†^
Morishita, K., 2014 [2]	3	1	10	6	3600	3.100	2.670 ^†^	1.870 ^†^
Oh, S., 2024 [3]	3	0.1	5	4.9	147	1.090	0.942 ^†^	0.517 ^†^
Oshikoya, C.A., 2000 [17]	1	1.5	7	4.1	2583	4.985 ^†^	3.630 ^†^	3.660
Rimington, S.J., 1995 [19]	1	1.5	10	10	9000	4.985 ^†^	2.000	2.955 ^†^
Weaver, S.L., 2006 [28]	1	1.5	10	5	4500	7.458 ^†^	5.100	2.955 ^†^
Draper, D.O., 2010 [24]	3	1	10	4.1	2460	13.300	9.095 ^†^	6.220 ^†^
Low Intensity Continuous Ultrasound								
Hendren, T.F., 2023 [1]	3	0.132	240	5	19,720	11.780	8.056 ^†^	5.509 ^†^
Langer, M.D., 2017 [6]	3	0.132	240	5	9504	11.500	7.864 ^†^	5.378 ^†^
Oh, S., 2024 [3]	3	0.1	60	4.9	1764	2.080	1.422 ^†^	0.973 ^†^
Rigby, J.H., 2015 [7]	3	0.132	180	5	14,000	4.450	3.180	2.081 ^†^
Scanzuso, A., 2025 [27]	3	0.132	60	5	4675	3.859 ^†^	2.639 ^†^	1.805 ^†^

^†^ Value calculated using stepwise regression model.

**Table 3:** Thermal effects of low-intensity continuous ultrasound (LICUS) and traditional ultrasound (US) at different depths.

Tissue Depth	Delivery Mode	Mean Duration of Treatment	Mean Temp Increase (°C, Mean ± SD)	*p*-Value
1 cm	LICUS	156 ± 91 min	6.84 ± 3.84	<0.001
	Traditional US	9 ± 3 min	5.24 ± 2.24	
3 cm	LICUS	156 ± 91 min	4.70 ± 2.62	<0.001
	Traditional US	9 ± 3 min	3.53 ± 1.65	
5 cm	LICUS	156 ± 91 min	3.20 ± 1.80	<0.001
	Traditional	9 ± 3 min	2.57 ± 1.26	

## Data Availability

The original contributions presented in this study are included in the article/Supplementary Material. Further inquiries can be directed to the corresponding author.

## Supplementary Materials

The following supporting information can be downloaded at: https://www.mdpi.com/article/10.3390/medsci14060631/s1, Table S1: Search strings used for each database.

## Abbreviations

The following abbreviations are used in this manuscript:

US	Ultrasound
LICUS	Low-Intensity Continuous Ultrasound
PRISMA	Preferred Reporting Items for Systematic Reviews and Meta-Analyses
ERA	Effective Radiating Area
RoB	Risk of Bias
ROBINS-I	Risk of Bias in Non-randomized Studies of Interventions
ANOVA	Analysis of Variance
LSD	Least Significant Difference
