# Supplementary material for: Low-Intensity Continuous Ultrasound vs. Traditional Ultrasound: A Systematic Review and Descriptive Analysis on Past and Future Gold Standards for Deep-Tissue Diathermy in Clinical Medicine
Source: Med Sci (Basel). 2026 Oct 2;14(6):631. doi: 10.3390/medsci14060631 (PMC13707125; doi:10.3390/medsci14060631)
Supplement: Supplementary file 1 [file medsci-14-00631-s001.zip › medsci-4485495-supplementary.pdf]

Supplementary Table S1. Search strings used for each database

Embase	('ultrasound therapy'/exp OR 'therapeutic ultrasound':ti,ab,kw OR 'ultrasound treatment':ti,ab,kw OR 'ultrasound therapy':ti,ab,kw OR ((ultrasound NEAR/3 therap*):ti,ab,kw) OR 'therapeutic ultrasonography':ti,ab,kw OR 'low-intensity ultrasound':ti,ab,kw OR 'low intensity ultrasound':ti,ab,kw OR 'low-intensity continuous ultrasound':ti,ab,kw OR 'low intensity continuous ultrasound':ti,ab,kw OR 'continuous ultrasound':ti,ab,kw OR ((continuous NEAR/3 ultrasound):ti,ab,kw) OR 'continuous therapeutic ultrasound':ti,ab,kw OR 'ultrasound diathermy':ti,ab,kw OR 'ultrasonic diathermy':ti,ab,kw OR 'acoustic medicine':ti,ab,kw OR 'li*us':ti,ab,kw<br>) AND (<br>'diathermy'/exp OR diatherm*:ti,ab,kw OR 'diathermic effect*':ti,ab,kw OR 'thermal effect*':ti,ab,kw OR 'tissue heat*':ti,ab,kw OR 'deep heat*':ti,ab,kw OR 'temperature'/exp OR 'body temperature'/exp OR temperatur*:ti,ab,kw OR 'temperature change*':ti,ab,kw OR 'temperature increase*':ti,ab,kw OR 'temperature rise':ti,ab,kw OR 'temperature elevation':ti,ab,kw OR 'tissue temperature':ti,ab,kw OR 'intramuscular temperature':ti,ab,kw OR 'muscle temperature':ti,ab,kw OR heating:ti,ab,kw OR warming:ti,ab,kw OR ((temperature NEAR/3 change):ti,ab,kw)<br>) AND (<br>'soft tissue'/exp OR 'muscle'/exp OR 'connective tissue'/exp OR 'soft tissue*':ti,ab,kw OR 'soft-tissue*':ti,ab,kw OR muscl*:ti,ab,kw OR intramuscular:ti,ab,kw OR tendon*:ti,ab,kw OR ligament*:ti,ab,kw OR 'connective tissue':ti,ab,kw OR 'deep tissue':ti,ab,kw OR 'calf':ti,ab,kw OR 'forearm':ti,ab,kw<br>) NOT 'focused ultrasound'
PubMed	("Ultrasonic Therapy"[Mesh] OR "therapeutic ultrasound"[tiab] OR "ultrasound therapy"[tiab] OR "ultrasound treatment"[tiab] OR "therapeutic ultrasonography"[tiab] OR (ultrasound[tiab] AND therap*[tiab]) OR "low-intensity ultrasound"[tiab] OR "low intensity ultrasound"[tiab] OR "low-intensity continuous ultrasound"[tiab] OR "low intensity continuous ultrasound"[tiab] OR "continuous ultrasound"[tiab] OR ("continuous"[tiab] AND ultrasound[tiab]) OR "continuous therapeutic ultrasound"[tiab] OR "ultrasound diathermy"[tiab] OR "ultrasonic diathermy"[tiab] OR "acoustic medicine"[tiab] OR "sustained acoustic medicine"[tiab] OR LITUS[tiab] OR LICUS[tiab]<br>) AND (<br>"Diathermy"[Mesh] OR "Body Temperature"[Mesh] OR “Temperature"[Mesh] OR diatherm*[tiab] OR "diathermic effect*"[tiab] OR "thermal effect*"[tiab] OR "tissue heat*"[tiab] OR "deep heat*"[tiab]<br>) AND (<br>temperatur*[tiab] OR "temperature change*"[tiab] OR "temperature increase*"[tiab] OR "temperature rise"[tiab] OR "temperature elevation"[tiab] OR "tissue temperature"[tiab] OR "intramuscular temperature"[tiab] OR "muscle temperature"[tiab] OR heating[tiab] OR warming[tiab] OR (“temperature change*”[tiab])<br>) AND (<br>"Soft Tissue"[Mesh] OR "Muscles"[Mesh] OR "Connective Tissue"[Mesh] OR "soft tissue*"[tiab] OR "soft-tissue*"[tiab] OR muscl*[tiab] OR intramuscular[tiab] OR tendon*[tiab] OR ligament*[tiab] OR "connective tissue"[tiab] OR "deep tissue"[tiab] OR calf[tiab] OR forearm[tiab]<br>) NOT "High-Intensity Focused Ultrasound Ablation"[Mesh]

Web of Science	TS=("therapeutic ultrasound" OR "ultrasound therapy" OR "ultrasound treatment" OR (ultrasound NEAR/3 therap*) OR "therapeutic ultrasonography" OR "low-intensity ultrasound" OR "low intensity ultrasound" OR "low-intensity continuous ultrasound" OR "low intensity continuous ultrasound" OR "continuous ultrasound" OR (continuous NEAR/3 ultrasound)<br>OR "continuous therapeutic ultrasound" OR "continuous mode ultrasound"<br>OR "continuous mode therapeutic ultrasound" OR "ultrasound diathermy"<br>OR "ultrasonic diathermy" OR "acoustic medicine" OR "sustained acoustic medicine" OR LITUS OR LICUS OR ultrasonotherap*<br>)<br>AND<br>TS=(diatherm* OR "diathermic effect*" OR "thermal effect*" OR "tissue heat*" OR "deep heat*" OR temperatur* OR "temperature change*" OR "temperature increase*" OR "temperature rise" OR "temperature elevation"<br>OR "tissue temperature" OR "intramuscular temperature" OR "muscle temperature" OR heating OR warming OR (temperature NEAR/3 change*) OR "heat generation" OR "heat transfer" OR "thermal profile" OR "thermal distribution"<br>)<br>AND<br>TS=("soft tissue*" OR "soft-tissue*" OR muscl* OR intramuscular OR tendon* OR ligament* OR "connective tissue" OR fascia OR "skeletal muscle" OR "adipose tissue" OR "subcutaneous tissue" OR "deep tissue" OR calf OR forearm<br>)<br>NOT<br>TS=("high intensity focused ultrasound")
